# Follicular Development and Secretion of Ovarian Hormones during the Juvenile and Adult Reproductive Lives of the Myelin Mutant *taiep* Rat: An Animal Model of Demyelinating Diseases

**DOI:** 10.1155/2018/5718782

**Published:** 2018-09-16

**Authors:** L. P. Muñoz-de-la-Torre, J. R. Eguibar, C. Cortés, A. Ugarte, A. Trujillo

**Affiliations:** ^1^Instituto de Fisiología, Benemérita Universidad Autónoma de Puebla, 72570 Puebla, Mexico; ^2^Vicerrectoría de Investigación y Estudios de Posgrado, Benemérita Universidad Autónoma de Puebla, 72000 Puebla, Mexico; ^3^Facultad de Ciencias Biológicas, Benemérita Universidad Autónoma de Puebla, 72570 Puebla, Mexico

## Abstract

Infertility and reproductive problems have been reported in women with several neurological disorders, for example, demyelination. However, the physiology of such problems has remained unknown so far. The *taiep* rats are an animal neurological model that initially shows a hypomyelination followed by a progressive demyelination of the central nervous system. This animal has reproductive problems, and the aim of this work is to characterize the follicular development, secretion of ovarian hormones, and presence of noradrenaline in the ovaries of the female *taiep* rats in the juvenile and adult stages. The *taiep* rats have low body weight (approximately 19% less than that of SD rats), a delay of 4 days in the age of vaginal opening, and an irregularity in the estrous cycle by the absence or prolongation of some estral cycle stage. In the juvenile stage, we observed a decrease of approximately 44% in the total number of follicles with a 15% increase of atresia and an 80% decrease in the fluorescence intensity of catecholamines in the ovaries, with a 21% increment in plasma concentrations of testosterone. In the adult stage, we observed follicular cysts and a 50% decrease in fluorescence intensity of catecholamines in the ovaries, with changes in the secretion of ovarian hormones, an increase of 20 times in progesterone, and a decrement of a half in estradiol. The demyelination in *taiep* rats affects follicular development and steroidogenesis in the early stages of the animal's life, and this is maintained until adulthood.

## 1. Introduction

Infertility and reproductive problems have been reported in women with multiple sclerosis (MS), a demyelinating disease. MS is one of the most important demyelinating diseases. It is currently known that 70% of the cases of MS occur between 20 and 40 years of age, and that it has a higher prevalence in women than in men with a 3 : 1 ratio [1, 2]. Reproductive problems have been reported in women with MS, ranging from sexual dissatisfaction and the absence of orgasms [3, 4], to menstrual irregularity and hormonal and ovarian follicular development alterations [5, 6]. However, the relationship between the presence of ovarian change and demyelinating disease has not been clearly stablished.

It is well known that the functions of the ovary are regulated by gonadotropins secreted by the pituitary gland as a result of the stimulus from the hypothalamus by the gonadotropin-releasing hormone (GnRH). In the last five decades, there has been an accumulation of evidence demonstrating that, in addition to the regulation exerted by gonadotropins on the ovary, there are other types of signals coming from the autonomous innervation that participate in the regulation of gonadal functions [7]. The ovary receives and sends nervous information via the superior ovarian nerve, the ovarian plexus, and the vagus nerve [8, 9]. These signals include classic neurotransmitters such as noradrenaline and acetylcholine, as well as several neuropeptides, such as NPY, VIP, and SP [10–12].

In this work, we proposed the use of the *taiep* rat as an animal model of demyelinating disease. This rat mutant is the result of a spontaneous mutation obtained in the F5 generation of the Sprague–Dawley strain obtained at the Benemérita Universidad Autónoma de Puebla (Mexico) in 1989 [13]. The *taiep* rat is characterized by various motor symptoms such as tremor, ataxia, immobility, epilepsy, and paralysis of the hind limbs. The name *taiep* is the acronym of these symptoms [13, 14].

We now know that this subline has an initial hypomyelination followed by a progressive demyelination of the CNS due to a mutation in chromosome 9. In fact, now we know that it is tubulin [15, 16]. It has prominent alterations in the electrical properties of neurons and in the electrical properties of the synaptic transmission in the spinal cord in the postnatal period [17]. Due to its symptoms, the *taiep* rat represents an important experimental model; Foote [18] proposed in 2005 that it as an animal model of demyelinating diseases. It has been shown to present a generalized epilepsy absence crises type, making it an ideal animal model for the study of several demyelinating pathologies [14, 18].

For these reasons, the aim of this study was to characterize the follicular development, secretion of steroid hormones, and presence of nerve fibers in the ovary on *taiep* rats in two stages of the life of the animal: juvenile and adult. In this way, we intend to contribute to knowledge about the ovarian level affectations that may be happening in an animal model of demyelinating disease.

## 2. Materials and Methods

### 2.1. Animals

**Figure 1:**
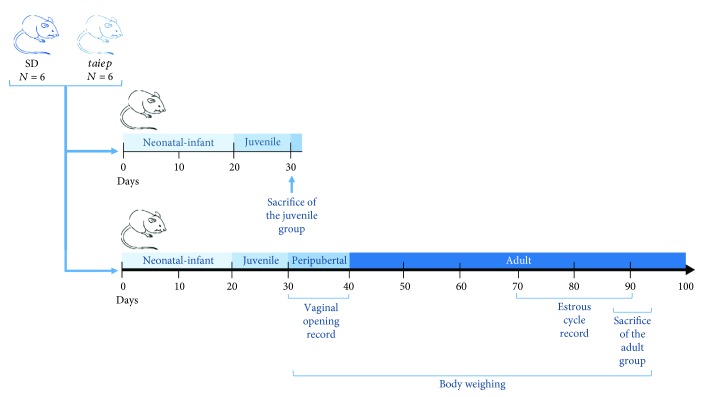
Experimental design. We used 6 *taiep* rats and 6 Sprague–Dawley (SD) rats divided into two groups: juvenile and adult. The rats in the juvenile group were sacrificed at the age of 30 days and those in the adult group were sacrificed in estrous at the age of 90 ± 5 days. Vaginal opening records, body weighing, and estrous cycle records were acquired in the adult group.

We maintained Sprague–Dawley and *taiep* rat groups in our animal room facilities under controlled 12:12 hr cycle (lights on 07:00), relative humidity (30–45%), and temperature (22 ± 2 °C) conditions. All experiments were performed in strict accordance with the Mexican Law of Animal Treatment and Protection Guidelines and the specifications of the Mexican Official Standard (NOM-062-ZOO-1999) for production, care, and use of laboratory animals that are in accordance with the National Institute of Health (NIH) guide.  Figure 1 shows the experimental design.

### 2.2. Assessment of the Vaginal Opening and Estrous Cycle

The juvenile animals were examined daily at 08:00 to recognize the vaginal opening. After the presence of vaginal canalization, the adult animals' estrous cycle was monitored daily for two weeks by vaginal lavage.

### 2.3. Autopsy

The juvenile group was sacrificed on the 30th day. The adult group was sacrificed at the age of 90 days (vaginal estrus preceded by proestrus). Animals were subjected to an autopsy between 08:00 and 10:00 hrs with overdose of pentobarbital. After, the trunk blood was obtained by decapitation in a soundproof and isolated room to avoid stress. Serum was collected and kept at room temperature for 30 minutes, and then centrifuged at 3500 rpm for 15 min. Serum was separated from cell buttons and stored at −20 °C in Eppendorf tubes until progesterone, 17*β*-estradiol, and testosterone quantifications were performed.

### 2.4. Steroid Hormone Quantification

Using the ELISA technique with commercial kits (DRG brand), we quantified the serum concentration of testosterone, estradiol, and progesterone. Kits for all hormones were used in accordance with manufacturer's instructions. The intra- and interassay variabilities for the estradiol kit used were less than 9% and 14%, respectively, with a detection limit of 2000 pg/mL. The intra- and interassay variabilities for the progesterone kit were less than 7% and 9%, respectively, with a detection limit of 40 ng/mL. The intra- and interassay variabilities for the testosterone kit were less than 4% and 9%, respectively, with a detection limit of 16 ng/mL. The optical density of the wells was measured at 450 nm with a universal microplate reader BioTek ELx800. Hormone concentrations were calculated using KCjunior software (BioTek Instruments).

### 2.5. Ovarian Morphology

Ovaries were fixed in Bouin's solution, dehydrated, and included in paraffin. Microtome sections 10 *μ*m thick were obtained and stained with hematoxylin–eosin (H3136-E4009 Sigma-Aldrich). The follicles present in a section every 100 *μ*m were classified according to their state as healthy or atretic. A follicle was considered atretic when it exhibited at least one of the following characteristics: the presence of oocyte with nuclear pycnosis, desquamation of the granulosa layers, and presence of fenestration in the granulosa layer. In addition, follicles were classified in relation to the thickness of the granulosa layer and the follicular diameter as follows: primary (P), secondary A (SA), secondary B (SB), preantral A (PA), preantral B (PB), antral A (AA), and antral B (AB), (Table 1). The criteria for reporting the presence of cysts are as follows: follicles that presented a wide antral cavity, a decrease of granulosa cell layer, thecal hyperplasia, and the absence of oocyte. The study of the follicular population was performed in the right and left ovaries of three animals per group taken randomly. Photographs of ovaries were taken every 100 *μ*m using a Moticam3 camera. Images were analyzed using the program ImageJ 1.50i (NIH).

### 2.6. SPG Method

The presence of catecholamines were visualized using the glyoxylic acid technique [19, 20]. Ovaries were obtained fresh and were cut in a cryostat (Leica CM1850) every 20 *μ*m. The cuts were treated with SPG solution (sucrose, potassium phosphate monobasic, and glyoxylic acid) and green malachite; then, they were dried with cold air and placed in an oven at 90 °C for 3 min. Subsequently, the cuts were assembled for observation under an epifluorescence or confocal microscope with an excitation of 450–490 nm. Thirty sections were analyzed per experimental and control groups. Images were analyzed in the program ImageJ 1.50i. In each section, we made a selection of 10,000 pixels around follicles to determine the intensity of the fluorescence using the histogram.

### 2.7. Statistical Analyses

Comparisons of more than two groups were analyzed with an ANOVA (multifactorial analysis of variance) followed by a Tukey's test. Student's *t*-test was used when we compare two groups. The Mann–Whitney *U* test was used for the follicular population analysis. Percentages were analyzed with a *z*-test. Differences in probabilities equal or less than 0.05 were considered statistically significant.

## 3. Results

### 3.1. Juvenile Stage

**Figure 2:**
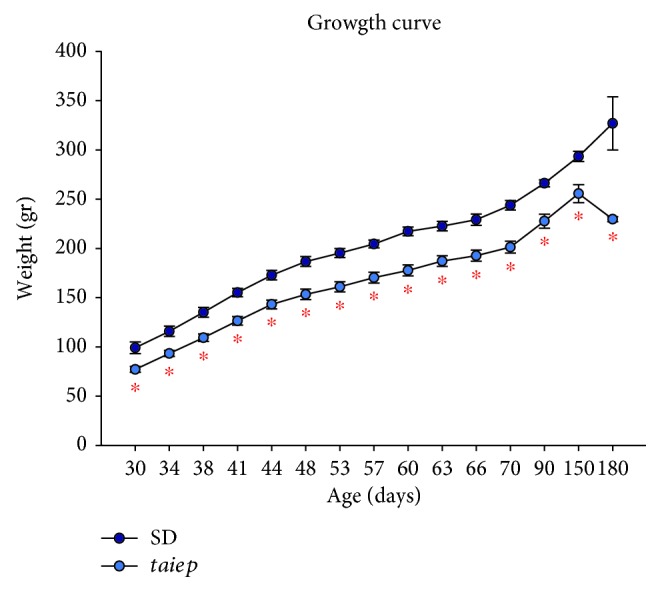
Plot of growth curve and weight (gr) with respect to age in days. Values are the mean and the error bars represent the standard error of measurements for 6 Sprague–Dawley rats (SD) or *taiep* rats in different ages of development. Comparisons were analyzed with an ANOVA followed by a Tukey's test, ^∗^*p* ≤ 0.001.

The body weight of *taiep* rats at the age of 30 days was 19% lower when compared to the body weight of Sprague–Dawley (SD) rats (77.2 ± 3 g of body weight vs. 99.2 ± 5.9 g of body weight, *p* < 0.05); this difference was preserved until the adult stage (Figure 2). A delay of 4 days in the vaginal opening of the *taiep* rat was observed (38.9 ± 0.3 days in *taiep* rat vs. 34 ± 0.2 days in SD rat; Student's *t*-test, *p* ≤ 0.001).

**Figure 3:**
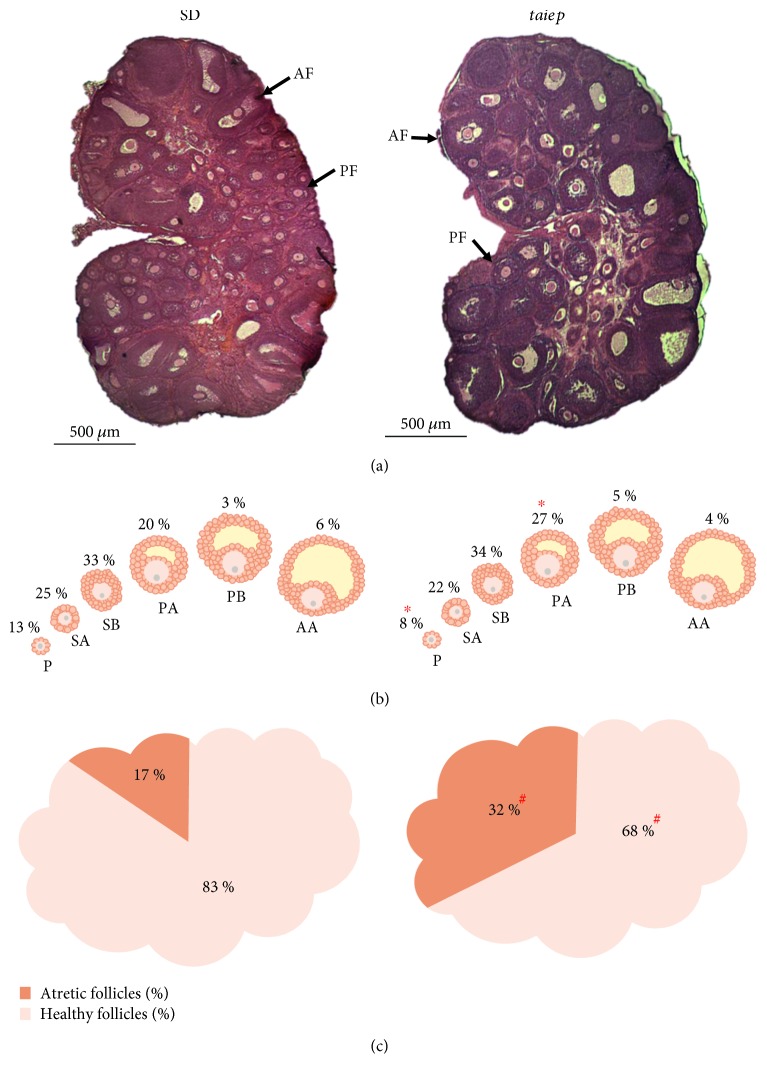
Follicle population in Sprague–Dawley and *taiep* rats in the juvenile stage. (a) Representative histological slice of the ovary. (b) Percentages of follicles in the different categories. (c) Percentages of total atretic and healthy follicles in the ovary. SD: Sprague–Dawley; AF: antral follicle; PF: preantral follicle; P: primary; SA: secondary A; SB: secondary B; PA: preantral A; PB: preantral B; AA: antral A; AB: antral B. ^∗^*p* ≤ 0.01*z*-test and ^#^*p* ≤ 0.001*z*-test.

The analysis of the ovaries showed a decrease of 44% in the total number of follicles present in the ovaries obtained from *taiep* in comparison to SD rats (334.33 ± 45.99 vs. 595 ± 17.04; Mann–Whitney *U* test with *p* ≤ 0.01) (Figure 3(a)). When analyzing the follicles present in the ovaries by each category, we observed that in the ovaries of the *taiep* rat there is a significant decrease in the percentage of P follicles and an increase in the percentage of PA follicles, in comparison with the follicles present in the ovaries of SD rats (Figure 3(b)).

Also, a decrease in the percentage of healthy follicles and an increase of 15% in atretic follicles were observed in the ovaries of *taiep* rats in comparison with SD rats (Figure 3(c)). When analyzing the follicles by category and stage (healthy or atretic), we observed that, with the exception of PB follicles, there was a decrease in the percentage of healthy follicles in all types of follicles present in the ovaries of the *taiep* rats when compared to SD rats (Table 2).

**Figure 4:**
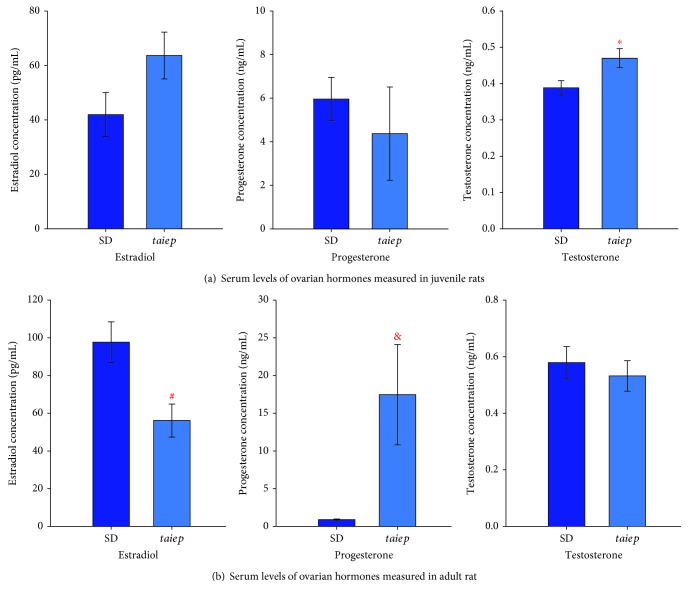
Serum levels of ovarian hormones in the juvenile and adult Sprague–Dawley and *taiep* rats. (a) Serum levels of estradiol, progesterone, and testosterone in the juvenile Sprague–Dawley and *taiep* rats. The error bars represent the standard error of measurements for 6 Sprague–Dawley rats (SD) or *taiep* rats. (b) Serum levels of estradiol, progesterone, and testosterone in adult Sprague–Dawley and *taiep* rats. The error bars represent the standard error of measurements for 6 Sprague–Dawley rats (SD) or *taiep* rats. SD: Sprague–Dawley. ^∗^*p* ≤ 0.033, ^#^*p* ≤ 0.009, and ^&^*p* ≤ 0.017; Student's *t*-test.

No significant differences were obtained in the serum levels of estradiol and progesterone, but there was an increase of 21% in the serum levels of testosterone observed in juvenile *taiep* with respect to SD rats (Figure 4(a)).

**Figure 5:**
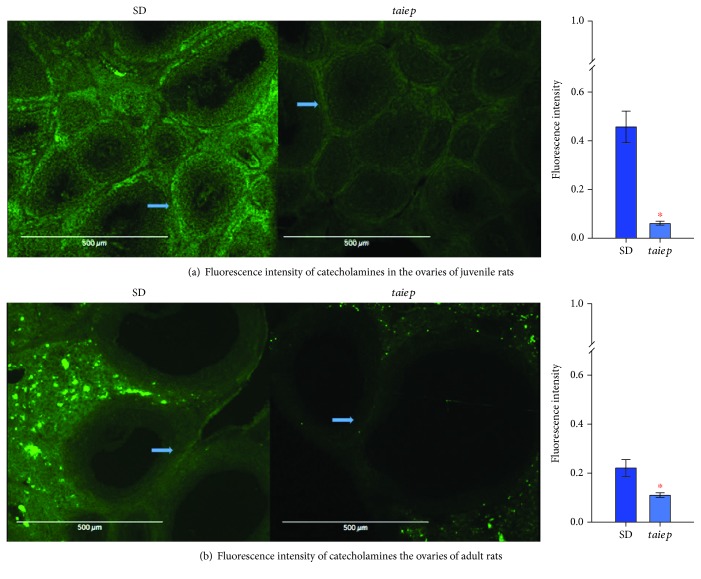
Fluorescence intensity of catecholamines in the ovaries of juvenile and adult Sprague–Dawley and *taiep* rats. (a) Immunohistochemistry of an ovary slice of juvenile Sprague–Dawley and *taiep* rats; arrows indicate the location of the fluorescent around the follicle; the bar graph shows fluorescence intensity. The error bars represent the standard error of measurements for 30 sections of ovaries by group. (b) Immunohistochemistry of an ovary slice of adult Sprague–Dawley and *taiep* rats; arrows indicate the location of the fluorescents, and the bar graph shows fluorescence intensity. SD: Sprague–Dawley. The error bars represent the standard error of measurements for 30 sections of ovaries by group. ^∗^*p* ≤ 0.01 Student's *t*-test.

Histochemical analyses revealed the presence of catecholamines in the ovaries of *taiep* and SD rats; these fibers were located preferentially in the periphery of the ovarian follicles, cortex, and corpora lutea. Determining fluorescence intensity analysis, we obtained a significant decrease of 80% in the fluorescence intensity of catecholaminergic fibers in the ovaries of *taiep* rats when compared to SD rats (Figure 5(a)).

### 3.2. Adult Stage

**Figure 6:**
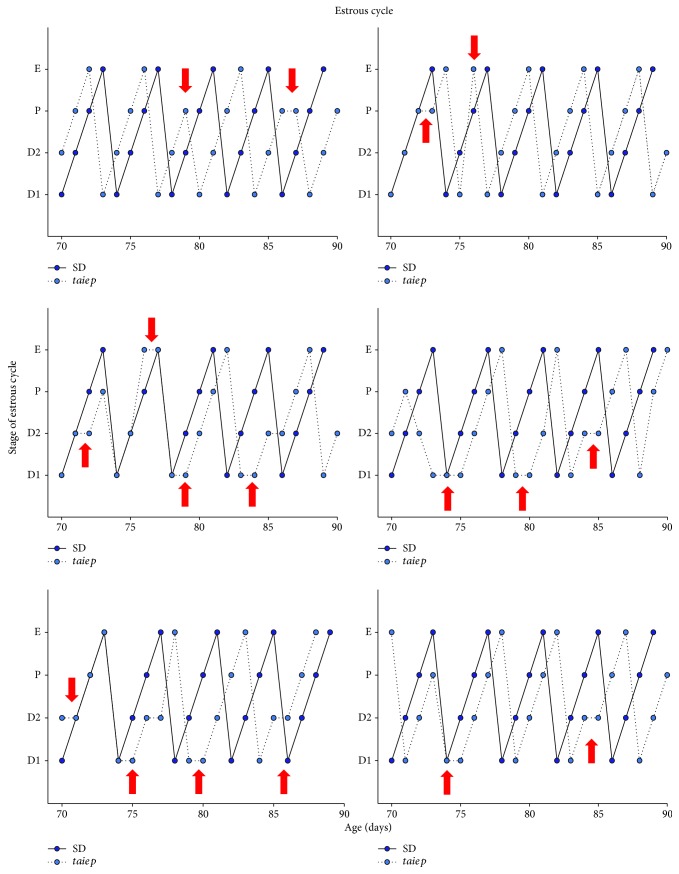
Estrous cycle of Sprague–Dawley and *taiep* rats from 70 days of age until sacrifice. Each graph represents the estrous cycle of one Sprague–Dawley and one *taiep* rat. The estrous cycle comprises four stages: estrus, diestrus 1, diestrus 2, and proestus. Red arrows indicate the moments when the estrous cycle of the *taiep* rat is irregular. SD: Sprague–Dawley; E: estrus; D1: diestrus 1; D2: diestrus 2; P: proestrus.

**Figure 7:**
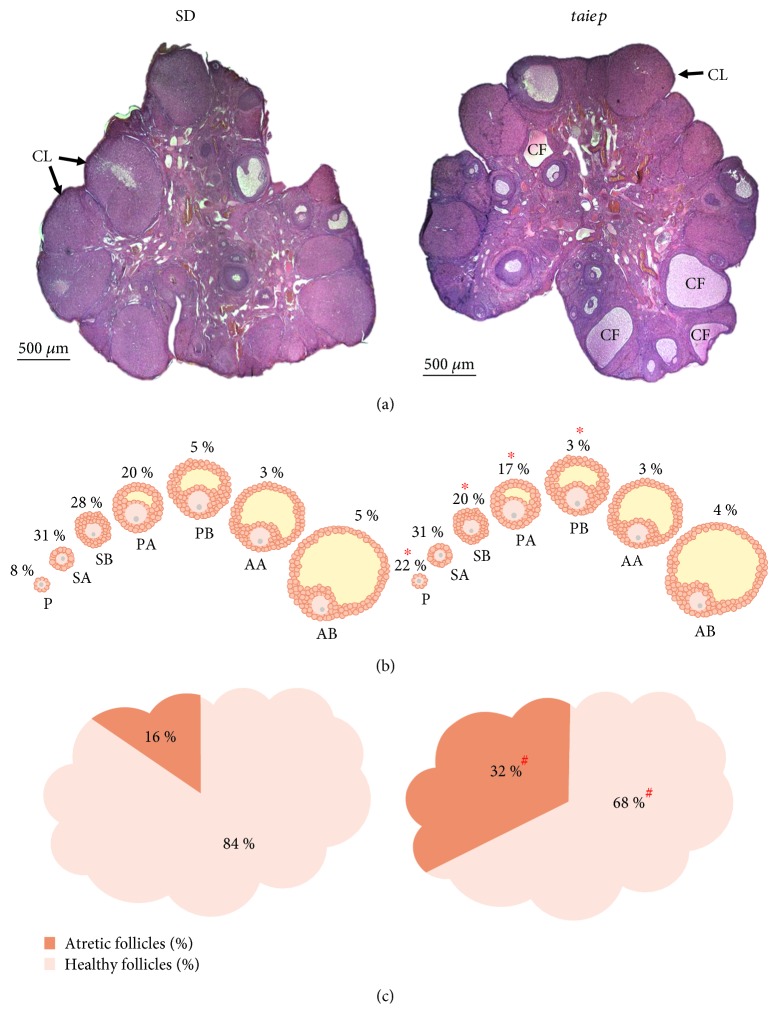
Follicle population in Sprague–Dawley and *taiep* rats in the adult stage. (a) Representative histological slice of the ovary. (b) Percentages of follicles in the different categories. (c) Percentages of atretic and healthy follicles in the ovary. SD: Sprague–Dawley; CF: cystic follicle; CL: corpora lutea; P: primary; SA: secondary A; SB: secondary B; PA: preantral A; PB: preantral B; AA: antral A; AB: antral B. ^∗^*p* ≤ 0.01*z*-test and ^#^*p* ≤ 0.001*z*-test.

Vaginal smears from 70 days of age show that *taiep* rats have irregular estrous cycles when compared to SD rats (Figure 6). Histological analyses revealed the presence of follicular cysts in the ovaries of *taiep* rats at 90 days of age (Figure 7(a)). There was no difference between both groups of rats in the total number of follicles present in the ovaries (*taiep*: 191.6 ± 22.0 vs. SD: 216 ± 25.3; Student's *t*-test, *p* ≤ 0.51). When analyzing the follicles present in the ovaries by category, we observed that in the ovaries of the adult *taiep* rats there were significant changes in the percentages of P, SB, PA, and PB follicles, in comparison with the follicles evaluated in the ovaries of SD rats (Figure 7(b)).

We observed a decrease in the percentage of healthy follicles and concomitantly an increase in the percentage of atretic follicles in the ovaries of *taiep* rats when compared to SD rats (Figure 7(c)). When analyzing the follicles by category and stage (healthy or atretic), we observed that, with the exception of P follicles, there was a decrease in the percentage of healthy follicles with respect to all types of follicle population present in the ovaries of *taiep* rats with respect to SD rats (Table 2).

In adulthood, the serum levels of estradiol decreased by half in *taiep* rats when compared to SD rats. The serum levels of progesterone were increased 20 times, and no significant differences were observed in the serum levels of testosterone (Figure 4(b)).

Histochemical analyses revealed the presence of noradrenergic fibers in the ovaries from *taiep* and SD rats, similar to what was observed in the ovaries from the juvenile stage. The fluorescence intensity analysis showed a decrease of 50% in the fluorescence intensity of catecholamines in the ovaries of the *taiep* rat that was maintained in adult life (Figure 5(b)).

## 4. Discussion

The present work yields relevant information related to the ovarian physiology on *taiep* rats, an important myelin mutant rat. The first finding is a decrease in body weight observed throughout the life span of the *taiep* rat, which is probably a consequence of the progressive demyelination of the central nervous system, but not in the peripheral system [21, 22]. A decrease in weight has been reported in animal models of demyelinating diseases, and in humans affected by Huntington's disease [23, 24]. Since the 1980s, it has been known that the beginning of the reproductive stage is affected by body weight, specifically, by the proportion of fat with respect to the whole body mass [25, 26]. The low weight of *taiep* rats may be one of the factors that result in the delay of the age of vaginal opening and the irregularity of their estrous cycles.

Another possible cause of the alteration of the estrous cycle in *taiep* rats is the high concentration of testosterone obtained by these rats at an early age. In Wistar rats, it has been reported that postnatal dihydrotestosterone administration delays vaginal opening and alters the estrous cycle [27]. In 21-day-old Sprague–Dawley rats given systemic testosterone, there is a delay in the age of vaginal opening and also an irregularity in the estrous cycle [28].

Given that a decrease in the follicular population and reserve has been reported in women with multiple sclerosis [6], in this study we characterized follicular development in the juvenile and adult stages of this myelin mutant.

During the juvenile stage, the *taiep* rat showed a significant decrease in the follicular population, with a decrease in the primary follicles and an increase in the preantral follicles and follicular atresia. This may be a consequence of the increase in the plasma concentration of testosterone [29]. It has been reported that administration of testosterone in 23-day-old Sprague–Dawley rats results in an increase in follicular atresia [30]. When characterizing the adult stage, we observed an increase of primary follicles and a decrease of growing follicles, along with an increase in follicular atresia and the presence of follicular cysts. All changes can be due to the high concentrations of testosterone present in the juvenile stage or due to the dismyelinating process. In 21-day-old Wistar rats which do not present demyelination of the central nervous system, the implantation of dihydrotestosterone for 11–13 weeks produces an increase in follicular atresia and causes the development of follicular cysts [31]. In Wistar rats, it has been reported that postnatal administration of dihydrotestosterone affects follicular development in the adult stage, causing the increase of preantral follicles, decrease of antral follicles, and appearance of follicular cysts [27].

Hormonal stimuli are not the only important element for ovarian function, since the presence of adequate noradrenergic neurons and their axons has been demonstrated in the ovaries of Wistar rats as well as the presence of noradrenergic fibers in the ovaries of Sprague–Dawley rats [11]. The main source of ovarian noradrenaline comes from the superior ovarian nerve [8], which is well known for participating in the processes of steroidogenesis and folliculogenesis, and also for stimulating the expression of FSH receptors in primordial follicles [12, 32].

In *taiep* rats, we observed a decrease in the fluorescence of catecholaminergic fibers at the juvenile stage and in adult animals. These changes in catecholaminergic fiber innervation may be due to the progressive demyelinating process that the *taiep* rat experiences. It has been reported that noradrenaline levels at the central level are altered in patients with multiple sclerosis and in animal models with experimental autoimmune encephalomyelitis, a model of MS which presents damage at the locus coeruleus, causing an increase of inflammation that can induce neuronal damage [33]. A decrease in noradrenaline in peripheral blood mononuclear cells of patients with MS has also been observed when the disease is inactive and when norepinephrine levels are increased, causing failure in apoptosis [34].

In *taiep* rats, the activation of astrocytes, along with the activation of the glial fibrilar acidic protein [22, 35] and concomitantly the activation of nitric oxide synthase, has been demonstrated to induce neuronal damage and endothelial dysfunction, which are probably due to a neuroinflammation process [35]. There is also an increased production of some chemokines [36] supporting an active inflammatory process that adds to progressive demyelination [22].

## 5. Conclusions

The results of the present work show alterations in ovarian functions in two stages of the life of the *taiep* rat, an animal model of demyelinating disease. These results allow us to conclude that demyelination at the level of the central nervous system in *taiep* rats affects follicular development and steroidogenesis in the early stages of the animal's life, and this is maintained until adulthood. Our results clearly contribute to the knowledge about the ovarian level affectations that may be happening in an animal model of demyelinating disease. This allows us to postulate that the *taiep* rat is a good animal model for continuing the analysis of alterations in ovarian physiology in animals with central demyelination. These studies will help in proposing possible reproductive strategies to improve fertility and ovarian function in women who suffer such diseases.

## Figures and Tables

**Table 1:** Classification of follicles in the base of morphological criteria.

Initials	Follicle	Granulosa layer thickness (*μ*m)	Follicular diameter (*μ*m)	Ideal morphology
P	Primary	<20	<100	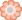
SA	Secondary A	20–30	100–150	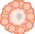
SB	Secondary B	30–40	150–200	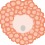
PA	Preantral A	40–60	200–300	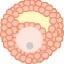
PB	Preantral B	60–80	300–400	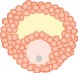
AA	Antral A	60–90	400–500	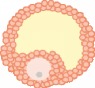
AB	Antral B	60–100	>500	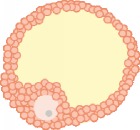

**Table 2:** Follicle population in the ovaries from the juvenile and adult *taiep* and Sprague–Dawley rats.

Juvenile	Total number of follicles	P (%)		SA (%)		SB (%)		PA (%)		PB (%)		AA (%)			
		A	H	A	H	A	H	A	H	A	H	A	H		
SD	595.00 ± 17.04	93	7	88	12	83	17	74	26	70	30	85	15		
*taiep*	334.33 ± 45.99 ^∗^	87^$^	13^$^	74^Δ^	26^Δ^	63^Δ^	37^Δ^	62^Δ^	38^Δ^	71	29	73^Δ^	27^Δ^		

Adult	Total number of follicles	P (%)		SA (%)		SB (%)		PA (%)		PB (%)		AA (%)		AB (%)	
		A	H	A	H	A	H	A	H	A	H	A	H	A	H
SD	216.00 ± 25.33	98	2	95	5	85	15	66	34	74	26	95	5	74	26
*taiep*	191.67 ± 22.06	96	4	86^#^	14^#^	43^Δ^	57^Δ^	42^Δ^	58^Δ^	33^Δ^	67^Δ^	50^Δ^	50^Δ^	33^Δ^	76^Δ^

SD: Sprague–Dawley; P: primary; SA: secondary A; SB: secondary B; PA: preantral A; PB: preantral B; AA: antral A; AB: antral B; A: atretic follicles; H: healthy follicles. ^∗^*p* ≤ 0.01 Mann–Whitney *U*; for $ *p* ≤ 0.004*z*-test, for # *p* ≤ 0.005*z*-test, and for Δ *p* ≤ 0.001*z*-test.

## Data Availability

The data used to support the findings of this study are available from the corresponding author upon request.

## References

[1] Rowan C. (2002). *Esclerosis múltiple: Esperanza en la investigación*.

[2] Bove R., Chitnis T. (2013). Sexual disparities in the incidence and course of MS. *Clinical Immunology*.

[3] Merghati-Khoei E., Qaderi K., Amini L., Korte J. E. (2013). Sexual problems among women with multiple sclerosis. *Journal of the Neurological Sciences*.

[4] Çelik D. B., Poyraz E. Ç., Bingöl A., İdiman E., Özakbaş S., Kaya D. (2013). Sexual dysfunction in multiple sclerosis: gender differences. *Journal of the Neurological Sciences*.

[5] Cavalla P., Rovei V., Masera S. (2006). Fertility in patients with multiple sclerosis: current knowledge and future perspectives. *Neurological Sciences*.

[6] Sepúlveda M., Ros C., Martínez-Lapiscina E. H. (2016). Pituitary-ovary axis and ovarian reserve in fertile women with multiple sclerosis: a pilot study. *Multiple Sclerosis Journal*.

[7] Morales L. L., Eguibar C. J. R., Cortes M. C., Trujillo H. A., Bonilla G. E. (2015). Regulación neuroendócrina del ovario. *Procesos fisiológicos y toxicológicos de la reproducción*.

[8] Aguado L. I. (2002). Role of the central and peripheral nervous system in the ovarian function. *Microscopy Research and Technique*.

[9] Gerendai I., Kocsis K., Halász B. (2002). Supraspinal connections of the ovary: structural and functional aspects. *Microscopy Research and Technique*.

[10] Hulshof S. C. J., Dijkstra G., van der Beek E. M. (1994). Immunocytochemical localization of vasoactive intestinal peptide and neuropeptide Y in the bovine ovary. *Biology of Reproduction*.

[11] D'Albora H., Lombide P., Ojeda S. R. (2000). Intrinsic neurons in the rat ovary: an immunohistochemical study. *Cell and Tissue Research*.

[12] Rosas G., Ramírez M. I., Linares R., Trujillo A., Domínguez R., Morales-Ledesma L. (2015). Asymmetric steroidogenic response by the ovaries to the vasoactive intestinal peptide. *Endocrine*.

[13] Holmgren B., Urbá-Holmgren R., Riboni L., Vega-SaenzdeMiera E. (1989). Sprague Dawley rat mutant with tremor, ataxia, tonic immobility episodes, epilepsy and paralysis. *Laboratory Animal Science*.

[14] Eguibar J. R., Cortés M. C. (2010). El mutante de mielina taiep como un modelo de crisis de ausencia. *Gaceta médica de México*.

[15] Li F.-Y., Song J., Duncan I. D. (2003). Mapping of *taiep* rat phenotype to rat Chromosome 9. *Mammalian Genome*.

[16] Duncan I. D., Bugiani M., Radcliff A. B. (2017). A mutation in the *Tubb4a* gene leads to microtubule accumulation with hypomyelination and demyelination. *Annals of Neurology*.

[17] Fuenzalida M., Roncagliolo P., Bonansco C., Roncagliolo M. (2004). Immature developmental pattern of the monosynaptic reflex in isolated spinal cord of glial mutant *taiep* rats. *Developmental Brain Research*.

[18] Foote A. K. (2005). Inflammation stimulates remyelination in areas of chronic demyelination. *Brain*.

[19] de la Torre J. C., Surgeon J. W. (1976). A methodological approach to rapid and sensitive monoamine histofluorescence using a modified glyoxylic acid technique: the SPG method. *Histochemistry*.

[20] Guidry G. (2016). A method for counterstaining tissues in conjunction with the glyoxylic acid condensation reaction for detection of biogenic amines. *Journal of Histochemistry & Cytochemistry*.

[21] Duncan I. D., Lunn K. F., Holmgren B., Urba-Holmgren R., Brignolo-Holmes L. (1992). The *taiep* rat: a myelin mutant with an associated oligodendrocyte microtubular defect. *Journal of Neurocytology*.

[22] Eguibar J. R., Cortes C., Ugarte A., León-Chávez A. (2014). The myelin mutant rat *taiep* as a model of neuroimmunological disease. *Advances in Neuroimmune Biology*.

[23] Aziz N. A., van der Burg J. M. M., Landwehrmeyer G. B. (2008). Weight loss in Huntington disease increases with higher CAG repeat number. *Neurology*.

[24] Djousse L., Knowlton B., Cupples L. A., Marder K., Shoulson I., Myers R. H. (2002). Weight loss in early stage of Huntington’s disease. *Neurology*.

[25] Frisch R. E. (1984). Body fat, puberty and fertility. *Biological Reviews*.

[26] Baker E. R. (1985). Body weight and the initiation of puberty. *Clinical Obstetrics and Gynecology*.

[27] Anesetti G., Chávez-Genaro R. (2016). Ovarian follicular dynamics after aromatizable or non aromatizable neonatal androgenization. *Journal of Molecular Histology*.

[28] Kim H. S., Shin J.-H., Moon H. J. (2002). Evaluation of the 20-day pubertal female assay in Sprague-Dawley rats treated with DES, tamoxifen, testosterone, and flutamide. *Toxicological Sciences*.

[29] Kimura S., Matsumoto T., Matsuyama R. (2007). Androgen receptor function in folliculogenesis and its clinical implication in premature ovarian failure. *Trends in Endocrinology & Metabolism*.

[30] Hillier S. G., Ross G. T. (1979). Effects of exogenous testosterone on ovarian weight, follicular morphology and intraovarian progesterone concentration in estrogen-primed hypophysectomized immature female rats. *Biology of Reproduction*.

[31] Mannerås L., Cajander S., Holmäng A. (2007). A new rat model exhibiting both ovarian and metabolic characteristics of polycystic ovary syndrome. *Endocrinology*.

[32] Malamed S., Gibney J. A., Ojeda S. R. (1992). Ovarian innervation develops before initiation of folliculogenesis in the rat. *Cell & Tissue Research*.

[33] Polak P. E., Kalinin S., Feinstein D. L. (2011). Locus coeruleus damage and noradrenaline reductions in multiple sclerosis and experimental autoimmune encephalomyelitis. *Brain*.

[34] Cosentino M., Zaffaroni M., Marino F. (2002). Catecholamine production and tyrosine hydroxylase expression in peripheral blood mononuclear cells from multiple sclerosis patients: effect of cell stimulation and possible relevance for activation-induced apoptosis. *Journal of Neuroimmunology*.

[35] Alicia Leon-Chavez B., Aguilar-Alonso P., Antonio Gonzalez-Barrios J. (2006). Increased nitric oxide levels and nitric oxide synthase isoform expression in the cerebellum of the *taiep* rat during its severe demyelination stage. *Brain Research*.

[36] Soto-Rodriguez G., Gonzalez-Barrios J.-A., Martinez-Fong D. (2015). Analysis of chemokines and receptors expression profile in the myelin mutant *taiep* rat. *Oxidative Medicine and Cellular Longevity*.

